# Prevalence and Risk Factors for Lymphocytic Choriomeningitis Virus Infection in Continental Croatian Regions

**DOI:** 10.3390/tropicalmed6020067

**Published:** 2021-04-29

**Authors:** Tatjana Vilibic-Cavlek, Tena Oreski, Misa Korva, Branko Kolaric, Vladimir Stevanovic, Snjezana Zidovec-Lepej, Irena Tabain, Pavle Jelicic, Bozana Miklausic-Pavic, Vladimir Savic, Ljubo Barbic, Tatjana Avsic-Zupanc

**Affiliations:** 1 Department of Virology, Croatian Institute of Public Health, 10000 Zagreb, Croatia; tatjana.vilibic-cavlek@hzjz.hr (T.V.-C.); irena.tabain@hzjz.hr (I.T.); 2 Department of Microbiology, School of Medicine, University of Zagreb, 10000 Zagreb, Croatia; 3 Gensynthese, Eurofins Genomics, 85560 Ebersberg, Germany; tenna.oreski@gmail.com; 4 Faculty of Medicine, Institute of Microbiology and Immunology, University of Ljubljana, 1000 Ljubljana, Slovenia; misa.korva@mf.uni-lj.si (M.K.); tatjana.avsic@mf.uni-lj.si (T.A.-Z.); 5 Department of Public Health Gerontology, Andrija Stampar Teaching Institute of Public Health, 10000 Zagreb, Croatia; branko.kolaric@stampar.hr; 6 Department of Epidemiology, Medical Faculty, University of Rijeka, 51000 Rijeka, Croatia; 7 Department of Microbiology and Infectious Diseases with Clinic, Faculty of Veterinary Medicine, University of Zagreb, 10000 Zagreb, Croatia; vladostevanovic@gmail.com (V.S.); ljubo.barbic@vef.hr (L.B.); 8 Department of Immunological and Molecular Diagnostics, University Hospital for Infectious Diseases “Dr Fran Mihaljevic”, 10000 Zagreb, Croatia; 9 Environmental Health Department, Croatian Institute of Public Health, 10000 Zagreb, Croatia; pavle.jelicic@hzjz.hr; 10 Department for General Infectious Diseases with Day Hospital, University Hospital for Infectious Diseases “Dr Fran Mihaljevic”, 10000 Zagreb, Croatia; bozana.miklausic@gmail.com; 11 Poultry Center, Croatian Veterinary Institute, 10000 Zagreb, Croatia; v_savic@veinst.hr

**Keywords:** lymphocytic choriomeningitis virus, seroprevalence, general population, professionally exposed, pregnant women, Croatia

## Abstract

Lymphocytic choriomeningitis virus (LCMV) is a neglected human pathogen associated with aseptic meningitis, severe systemic infections in immunocompromised persons, and congenital anomalies. Data on the prevalence of LCMV infections are scarce. We analyzed the seroprevalence of LCMV in continental Croatian regions. A total of 338 serum samples of professionally exposed (forestry workers, hunters, agriculture workers in contact with rodents) and non-exposed populations (general population, pregnant women) were tested for the presence of LCMV antibodies using indirect immunofluorescence assay. No participants reported recent febrile disease. LCMV IgG antibodies were detected in 23/6.8% of participants: 9.8% exposed persons and 5.1% non-exposed persons (6.1% in the general population and 3.9% in pregnant women). No participants were LCMV IgM positive. Although higher seropositivity was found in males compared to females (8.9% vs. 4.7%), inhabitants of suburban/rural areas compared to inhabitants of urban areas (9.2% vs. 4.6%), and persons who used well as a source of water compared to those who used tap (11.4% vs. 5.6%), these differences did not reach statistical significance. Results of logistic regression showed that the presence of rodents in the house/yard and cleaning rodent nests were associated with an elevated risk for LCMV infection (OR = 2.962, 95% CI = 1.019–8.607).

## 1. Introduction

Lymphocytic choriomeningitis virus (LCMV) is a neglected zoonotic virus associated with aseptic meningitis in immunocompetent individuals, life-threatening systemic infections in immunocompromised patients as well as spontaneous abortions in pregnant women and severe congenital infections [1]. LCMV is a rodent-borne virus that belongs to the *Arenaviridae* family, genus *Mammarenavirus*. The natural rodent host and principal reservoir for LCMV is the house mouse (*Mus musculus*, *Mus domesticus*), but antibodies were detected in some other rodent species such as the bank vole and yellow-necked mouse [2]. Pet hamsters may be competent alternative reservoirs. LCMV infections in rodents are often subclinical; however, rodents maintain high concentrations of virus in many organs [3]. Humans become infected by inhaling infectious aerosolized particles of dried rodent excreta, ingesting of food contaminated with the virus or by contamination of mucus membranes with infected body fluids [4]. Human-to-human transmission does not seem to occur, except through organ transplantation [5] and transplacental transmission from an infected mother to fetus [6].

The clinical spectrum of disease in immunocompetent individuals varies from asymptomatic infection or a mild self-limiting illness to aseptic meningitis and rarely, meningoencephalitis. Other reported neurological complications associated with LCMV include transverse myelitis, hydrocephalus and sensorineural hearing loss [4,7,8]. Rare non-neurological manifestations of LCMV infection such as pancreatitis, orchitis, arthritis, parotitis, and pericarditis have also been reported [4]. However, in immunocompromised patients such as transplant recipients, LCMV infection may show a different clinical course that resembles viral hemorrhagic fever. Several clusters of fatal transplant-associated LCMV infection with multiorgan failure have been described [9,10,11]. In contrast to adult infection in which severe disease is rare, LCMV infections in pregnant women often have a severe negative impact on the fetus [4]. Infection during the first trimester of pregnancy is associated with an increased risk of spontaneous abortion [12]. Infection during the second and third trimesters has been linked to congenital LCMV infection characterized by hydrocephalus, macrocephaly, or microcephaly, psychomotor retardation, periventricular calcifications, and chorioretinitis [13,14,15,16]. Approximately 35% of infants die from complications of congenital LCMV infection [4].

Because of the cosmopolitan distribution of its reservoirs, LCMV most likely circulates globally (except Antarctica) [1]. However, since many infections are asymptomatic or present as a mild self-limiting illness, the prevalence of clinically significant LCMV infection in humans is unknown and probably underestimated. Seroepidemiological studies revealed that up to 15% of the population is LCMV seropositive [17,18,19,20,21,22]. While some studies showed higher seroprevalence rates in specific population groups such as forestry workers or hunters, others did not find differences in the seropositivity between persons professionally exposed to rodents and the general population [23,24].

In Croatia, there are only two published studies on the seroprevalence of LCMV infection in humans conducted in 2006 in limited geographic areas and specific population groups. A study conducted among forestry workers in Posavina, a geographic region along the Sava River Basin, found a seropositivity of 5.1% [25]. The other study conducted in the rural population of Vir, a small island at the Croatian littoral, which is an endemic region for murine typhus, showed a very high seroprevalence rate of 36% [26]. Additionally, one study analyzed the presence of zoonotic viruses in rodents and small mammals trapped between 2003 and 2011. LCMV was detected in 0.8% of rodent samples from continental Croatia [27].

The aim of this study was to analyze the seroprevalence and risk factors for LCMV infection in exposed and non-exposed persons in the Croatian mainland.

## 2. Materials and Methods

### 2.1. Study Participants

During a one-year period (June 2016–May 2017), a total of 338 serum samples collected from professionally exposed and non-exposed persons in continental Croatian regions were tested for the presence of LCMV antibodies using indirect immunofluorescence assay (IFA). The exposed group consisted of 42 forestry workers, 36 hunters, and 44 agriculture workers who reported contacts with rodents in house/resting house (mean age 44.9 ± 11.1, range 20–72 years). The non-exposed group consisted of 115 subjects from the general population (mean age 45.6 ± 16.5, range 18–84 years) and 101 pregnant women (mean age 33.4 ± 4.5, range 20–44 years). Samples from the exposed population were collected during their regular systematic examinations at the Sv. Rok Polyclinic, while participants in the non-exposed group were volunteers coming to the Outpatient Department of the Croatian Institute of Public Health. Since pregnant women represent a specific population for LCMV infection because of possible transplacental transmission of virus to the fetus, they were analyzed as a separate group. All participants were asymptomatic at the time of testing and did not report recent febrile disease. After obtaining informed consent, all participants were interviewed to collect information regarding their sociodemographic characteristics (age, gender, education, occupation) and potential risk factors (place of residence, source of drinking water, contact with rodents, household animals). Data were collected using a modified Health-Environment-Life Style (HELS) questionnaire created by the Environmental Health Department, Andrija Stampar School of Public Health, University of Zagreb.

### 2.2. Geographic Features of the Study Area

Based on the geographic features of the studied area, two regions were defined: central (Zagreb macro-region) and eastern (Slavonia). The Zagreb area is an urban zone located on the slopes of the Medvednica Mountain (the highest peak is Sljeme which reaches an altitude of 1032 m). Zagreb surrounding areas (Hrvatsko Zagorje, Kordun, Banovina, Moslavina, and Prigorje) are formed of hills with many villages and small towns spread across the hillside. Slavonia (Baranja, Srijem) is a geographical region in eastern Croatia which is generally known as lowland, mostly up to 200 m above sea level. Mountains higher than 500 m are rare and of an insular character. A wide area of continental Croatia is covered by forests.

### 2.3. Serological Testing

**Figure 1:**
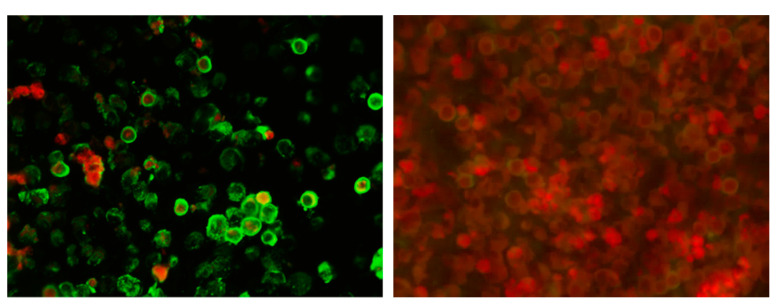
IFA-LCMV IgG positive sample (**left**), negative sample (**right**); 400× magnification. Evans Blue dye was used as a counterstain. Positive reaction is seen as apple-green fluorescence in the cytoplasm. Negative reaction is seen as red-counterstained cells.

Serological testing was performed using IFA (Figure 1). The assay was developed by modification of an already described method [19]. L929 cells infected with the LCMV Armstrong strain were used as antigen. After the cells reached 80% confluence, 1 mL of the virus was transferred to a 25 cm^2^ tissue culture flask and incubated at 37 °C with 5% of CO_2_ for 1 h. Cells in the control flask, at the same confluence level, were incubated with 1 mL of Eagle’s Minimum essential medium (MEM) (Capricorn Scientific GmbH, Ebsdorfergrund, Germany). After adding MEM that included 5% fetal calf serum (Capricorn Scientific GmbH, Ebsdorfergrund, Germany), flasks were kept at 37 °C with 5% of CO_2_ and checked daily for the cytopathic effect (CPE). At the first sign of CPE in infected cells, the supernatant was removed. In both flasks, cells were detached by trypsin. After cell counting, uninfected and infected cells were mixed in a ratio of 2:3 (to discriminate the nonspecific binding of antibodies to cells) to a final concentration of 1 × 10^6^ cells/mL and washed three times with phosphate-buffered saline (PBS). Cell suspensions were spotted onto 16-well glass slides at a volume of 10 µL per well, air-dried and stored at −20 °C until use. Ice cold acetone (Kemika, Zagreb, Croatia) was used for cell fixation. Successful infection and the presence of LCMV were confirmed in the supernatant of infected cell cultures using a reverse transcriptase-polymerase chain reaction (RT-PCR) [28]. Diluted serum samples were added to slides and incubated in a moist chamber at room temperature for 30 min. After washing of slides in PBS, three times and once with distilled water, anti-human IgG FITC conjugate containing Evans Blue as a counterstain for reducing background fluorescence (Vircell, Granada, Spain) was added and incubated for 30 min at room temperature. After washing, as above, the slides were examined independently by two researchers under a Leitz Laborlux fluorescence microscope. Serum samples were diluted to 1:16 in PBS for initial screening, and repeat testing with two-fold serial dilutions was performed on all positive samples to determine the endpoint titer. Positive and negative control serum samples were included in each test run. The intensity of fluorescence was compared with the positive control reaction. Patterns of reactivity different than those seen in the positive control were considered nonspecific. IFA titer ≥1:16 was considered positive [26]. In addition, IgG positive samples were further tested for the presence of IgM antibodies to confirm/rule out recent LCMV infection. All serum samples were pre-absorbed with anti-IgG antibodies (Eurosorb, Euroimmun, Lübeck, Germany) prior to testing for IgM antibodies. This prevents rheumatoid factor of IgM class present in the sample from reacting with specifically bound IgG (false positive IgM result), or specific IgG displacing IgM from antigen (false negative IgM result).

### 2.4. Statistical Analysis

LCMV seroprevalence rates were expressed as a percentage with 95% confidence intervals (CI). X^2^ or Fischer’s exact test were used to compare differences between groups. Strength of association between dependent variables (LCMV IgG positivity and a priori selected potential risk factors: age, gender, place of residence, educational level, water sources, food storage, contact with rodents, pet ownership) were assessed using logistic regression. Statistical analysis was performed using STATA/IC version 14.1 (StataCorp. LP, College Station, TX, USA). The level of statistical significance was α = 0.05.

## 3. Results

LCMV IgG antibodies were detected in 23/338; 6.8% (95% CI = 4.4–10.0) of participants, with seroprevalence rates of 9.8% (95% CI = 5.2–16.6) in exposed persons, 6.1% (95% CI = 2.5–12.1) in the general population, and 3.9% (95% CI = 5.2–16.6) in pregnant women (Table 1). LCMV IgG titers ranged from 1:16 to 1:128. No participants were LCMV IgM positive.

Prevalence of LCMV antibodies according to demographic data and potential risk factors is presented in Table 2. In addition to contact with rodents through work activities (professional exposure; 36.1%), many participants reported some other risk factors such as cat/dog ownership (51.2%), suburban/rural place of residence (48.2%), well as a source of drinking water (20.7%), food storage in the basement (17.2%), and the presence of rodents in their house/cleaning rodents’ nests (9.5%). According to age, the highest seropositivity (12.1%) was detected in the 41–50 year old group compared to 3.9–7.9% in the other age groups. Although higher seropositivity rates were found in males compared to females (8.9% vs. 4.7%) and persons with primary school compared to those with high school/university education (10.9% vs. 6.8%/4.6%), these differences were not significant. In addition, there was no difference in the seropositivity in inhabitants of suburban/rural areas compared to inhabitants of urban areas (9.2% vs. 4.6%), persons who used well as a source of water compared to those who used tap (11.4% vs. 5.6%), and participants who reported food storage in the basement compared to those who did not (8.6% vs. 6.4%). Furthermore, dog and cat ownership were not associated with the LCMV seroprevalence.

Results of logistic regression showed that the presence of rodents in the house/yard and cleaning rodent nests were significant predictors for LCMV seropositivity. Persons who reported the presence of rodents in house/yard and cleaning of the rodent nests showed almost three times higher risk for LCMV infection (OR = 2.962, 95% CI = 1.019–8.607) (Table 3).

**Figure 2:**
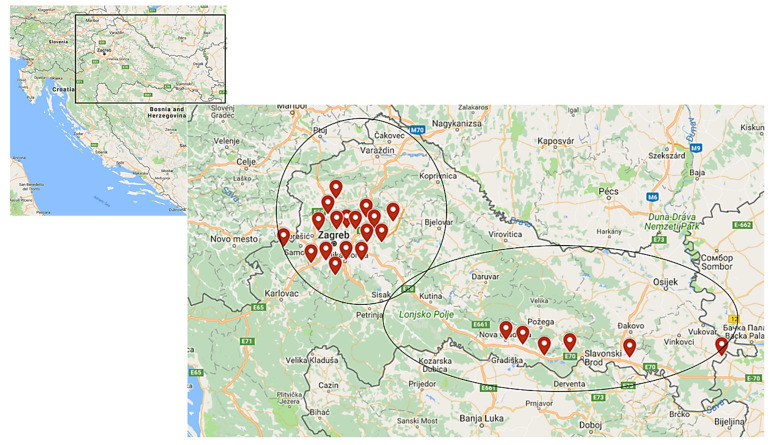
Geographic distribution of LCMV seropositive participants.

Geographic distribution of LCMV seropositive participants is presented in Figure 2. According to the geographical region, LCMV antibodies were detected in 21/266 (7.9%) of participants from central Croatian regions and 2/72 (2.8%) of participants from eastern Croatian regions.

## 4. Discussion

There are very few recently published serological surveys of LCMV infection. In this study, the overall seroprevalence rate was 6.8% which is similar to the seropositivity in an Iraqi study conducted in 2012–2013 (6.4%) [20] and an Italian study conducted in 2015 (7%) [29]. In the 1990s, several studies were conducted in the Americas. The reported seroprevalence rates in the general population were found to be 2.3% (1.54–6.06%) in Argentina [30], 2.4% in San Antonio (Texas) [31], 4% in Nova Scotia (Canada) [32], and 4.3–5.1% in Birmingham (Alabama) [31]. In the 2000s, seropositivity was reported to be 1.7% in Spain [17] and 3.3% in Argentina [18]. A very low seroprevalence rate was found in blood donors from Marseilles, France in 2007 (0.33%) [33] and blood donors from New York in 2009 (0.2%). Since blood donors were volunteers, the population tested did not necessarily reflect the population at risk for LCMV exposure [34]. A more recent study from Vietnam (2015) also showed a low LCMV seropositivity of 0.8% [21].

A significant increase in the seroprevalence of LCMV was documented in the province of Trento, Italy from 2.46% in 2002 to 7% in 2015 [29], but no risk factors including occupation and many outdoor activities such as gardening, having a woodshed, having a pet rodent, and collecting mushrooms were significantly correlated with this increase. The only factor that appeared to be more important was woodcutting [29].

Two studies analyzed the seroprevalence of LCMV in patients with neurological symptoms. A study from Finland conducted in 2013–2014 found 5.0% of patients were positive for LCMV IgG antibodies. Seropositivity was equally distributed between female and male patients, with the highest rate being in 5–10 year olds (16%), and from patients in the Helsinki and Uusimaa Hospital District (southern Finland). Seroprevalence was much lower in older age groups (1.8–4.0%) [22]. A recently published study from southern Iraq analyzed the seroprevalence of LCMV in patients with fever and neurologic manifestations and healthy persons (control group) tested during 2012–2016. The overall LCMV IgG seroprevalence was 8.8%. In the control group, 12.2% of participants were seropositive compared to 7% in the group of acute febrile patients [23]. Additionally, LCMV RNA was detected in 5.1% of cerebrospinal fluid samples from patients with a neuroinvasive infection in southern Iraq [20].

Several studies found that seroprevalence rate differs in some population groups with higher seropositivity in persons exposed to rodents [24,29]. Forestry workers, hunters, agricultural workers, and persons who reported frequently visiting forest areas represented exposed populations in our study. In this group, the LCMV seroprevalence was 9.8% compared to 5.1% in non-exposed groups (6.1% in the general population and 3.9% in pregnant women). A similar seroprevalence of 5.31% was reported in a low-risk group in Italy [29]. An Austrian study found that 13% employees of the zoological garden of Vienna, Schönbrunn were seropositive to LCMV [24]. In two studies conducted in northern Italy (the Province of Trento), seroprevalence among forestry workers was low in 2005 (2.5%) [19], but it was significantly higher in 2015 (8.02%). In addition, seroprevalence was high in hunters (12.9%) [29]. These results are similar with the seroprevalence of the exposed population in our study. In a Dutch study, pig farmers and veterinarians were tested for the presence of LCMV. The seroprevalence results showed LCMV antibodies in 2.6% of pig farmers, while none of the veterinarians were LCMV seropositive [35].

Although not significant, our results showed a difference in the seropositivity between genders with higher seroprevalence in men (8.9%) compared to women (4.7%). Men are more often in contact with rodents due to professional exposure (forestry workers, hunters, fishermen) which could explain this difference. However, a previously published Croatian study conducted among inhabitants of the Vir Island showed no gender difference [26]. In Argentina, significantly higher LCMV seroprevalence was found in men [18,23], while in Canada seroprevalence was higher in women [32]. The possible reason for this gender distribution is that women are more frequently exposed to dust contaminated with the excreta of house mice during the cleaning of the house and yard. In one Iraqi study (2012–2013) [20], a higher seroprevalence was found in women (9.7% vs. 3.5%), while in another one (2012–2016), men were more often seropositive compared to women (7.9% vs. 5.6%). However, in patients with acute febrile illness, the gender ratio was reversed (3.9% in women, 2.8% in men) [23].

This study showed the highest seroprevalence rate (12.7%) in the 41–50 year old age group compared to 3.1–7.9% in other groups. In a Croatian study conducted on the Vir Island, seropositivity was high in all age groups ranging from 32% to 40% [26]. In a study conducted in Iraq, an increase in seropositivity with age was found ranging from 1.6% in persons younger than 30 years to 10.3% in those older than 50 years [20]. A higher seropositivity in older people could be explained by longer exposure to the virus during the lifetime.

Our results showed that although not significantly, seropositivity was higher among inhabitants of suburban and rural areas compared to inhabitants of urban areas (9.2% vs. 4.6%). In contrast, in an Iraqi study seroprevalence was higher in urban areas (10.1% vs. 4.4%) [20], while a Spanish study found no difference in the seropositivity among residents of rural and urban areas [17]. Crowding in cities may explain the increased LCMV seropositivity among the urban population [20].

Similar to results of a study from Vietnam [21], our study showed no difference in seropositivity between persons owning a cat or dog and those who did not have household contacts with animals.

This study showed that contact with rodents in the house/yard and cleaning rodents nests correlated strongly with LCMV seroprevalence. Persons who reported these risk factors had an almost three times higher risk for LCMV seropositivity compared to non-exposed persons. A Dutch study compared the seroprevalence in active forestry workers (high-risk group) and supervisory forestry staff (low-risk group). Supervisory forestry staff had a three times higher risk, while active forestry workers had a five times higher risk for LCMV seropositivity compared to office workers (control group) [36]. A previously conducted study in Croatia among forestry workers showed that for one working year probability for LCMV seropositivity increased 9% [25].

Very few published studies analyzed the prevalence of LCMV in pregnant women. In Argentina, 1.6% of pregnant women were found to be LCMV positive, but the absence of LCMV antibodies in the newborns indicated that the mothers were infected before pregnancy [18]. In this study, 3.9% of pregnant women showed antibodies to LCMV; however, IgM antibodies were not detected suggesting previous LCMV infection. Since commercial serological assays for LCMV are not widely available, the number of congenital infections is probably underestimated. It is essential to emphasize that LCMV should be considered in the differential diagnosis for infants and children with unexplained hydrocephalus, micro- or macrocephaly, intracranial calcifications, chorioretinitis, and nonimmune hydrops [16,37].

LCMV seropositive participants were distributed in both central and eastern Croatian regions. The same geographic area is endemic for some other rodent-borne pathogens such as hantaviruses. Since 2002, sporadic hantavirus infections as well as large outbreaks (2012, 2014, 2017) are regularly reported in continental Croatian regions [38,39,40,41,42]. Hantaviruses Puumala (PUUV) and Dobrava (DOBV) were documented in humans, while Saaremaa virus and Tula virus were recorded in rodents [43,44,45,46]. In 2017, a seroprevalence study was conducted in exposed and non-exposed persons from continental Croatia. Seroprevalence rates of PUUV were 13.5% in forestry workers, 3.9% in hunters, and 2.5% in the general population. In addition, in 4.5% of forestry workers DOBV IgG antibodies were found. The highest seropositivity of both PUUV (28.2%) and DOBV (5.1%) was detected in agriculture workers who reported frequent contact with rodents [47]. The presented results indicate that endemic areas for LCMV and PUUV overlap.

The main limitation of this study is a small number of seropositive participants which might reduce the possibility to demonstrate significant differences for some potential risk factors and LCMV seropositivity. Therefore, the results should be interpreted with caution. Furthermore, IFA results are not confirmed using other serological methods. The plaque reduction neutralization test is the gold standard serological test that distinguishes different arenaviruses; however IFA is a very specific and widely used method for detection of both LCMV IgM and IgG antibodies [48]. Although limited by the small sample size, our results suggest that LCMV is present in Croatia and provide some new data on the epidemiology of this neglected viral zoonosis. Further studies on large samples are needed to confirm the role of LCMV in the etiology of neuroinvasive infections in the Croatian population. In addition, the detection of 3.9% seropositive pregnant women highlights the need for surveillance of LCMV as a potential cause of congenital infections.

## Figures and Tables

**Table 1:** Seroprevalence of LCMV in continental Croatia.

Population Group	N Tested (%)	LCMV IgG N (%)	95% CI
Overall	338 (100)	23 (6.8)	4.4–10.0
Exposed persons	122 (36.1)	12 (9.8)	5.2–16.6
General population	115 (34.0)	7 (6.1)	2.5–12.1
Pregnant women	101 (29.9)	4 (3.9)	1.1–9.8

**Table 2:** Prevalence of LCMV antibodies according to sociodemographic characteristics and risk factors.

Characteristic		N Tested (%)	N Positive (%)	95% CI	*p* Value
Population group	Exposed	122 (36.1)	12 (9.8)	5.2–16.6	0.116
	Non-exposed	216 (63.9)	11 (5.1)	2.6–8.9	
Gender	Male	169 (50.0)	15 (8.9)	4.6–13.5	0.338
	Female	169 (50.0)	8 (4.7)	2.5–9.9	
Age group	≤30 years	66 (19.5)	4 (6.1)	1.7–15.0	0.102
	31–40 years	127 (37.6)	5 (3.9)	0.9–7.9	
	41–50 years	56 (16.6)	7 (12.5)	5.3–22.4	
	50+ years	89 (26.3)	7 (7.9)	3.3–15.7	
Education	≤Elementary school	46 (13.6)	5 (10.9)	3.6–12.3	0.405
	High school	205 (60.7)	14 (6.8)	3.8–11.1	
	University	87 (25.8)	4 (4.6)	1.3–11.4	
Place of residence	Urban	175 (51.8)	8 (4.6)	2.0–8.8	0.129
	Suburban/rural	163 (48.2)	15 (9.2)	5.2–14.7	
Water source	Tap	268 (79.3)	15 (5.6)	3.2–9.1	0.107
	Well	70 (20.7)	8 (11.4)	5.1–21.3	
Food storage in basement	Yes	58 (17.2)	5 (8.6)	2.9–19.0	0.392
	No	280 (82.8)	18 (6.4)	3.9–10.0	
Contact with rodents *	Yes	32 (9.5)	5 (15.6)	5.3–32.8	0.054
	No	306 (90.5)	18 (5.9)	3.5–9.1	
Companion animals (dog, cat)	Yes	173 (51.2)	12 (6.9)	3.6–11.8	0.922
	No	165 (48.8)	11 (6.7)	3.4–11.6	

* Presence of rodents in house/yard, cleaning rodents’ nests.

**Table 3:** Logistic regression risk for LCMV IgG seropositivity.

Characteristic	OR	95% CI (OR)
Male vs. female gender	1.605	0.675−3.817
Age (one-year increase)	1.017	0.986–1.049
≤Elementary school education	1	
High school education	0.601	0.205–1.761
University education	0.395	0.100–1.550
Area of residence (suburban/rural vs. urban)	2.114	0.872–5.128
Food storage in basement (yes vs. no)	1.373	0.488–3.861
Water source (well vs. tap)	2.176	0.883–5.363
Presence of rodents in house/yard; cleaning rodents’ nests (yes vs. no)	2.962 *	1.019–8.607 *
Companion animals: cat/dog (yes vs. no)	1.043	0.447–2.435

* Statistical significance.

## Data Availability

Not applicable.
